# Opportunities and Challenges of Population Pharmacogenomics

**DOI:** 10.1111/ahg.12596

**Published:** 2025-04-02

**Authors:** Yitian Zhou, Yoomi Park, Mahamadou D. Camara, Volker M. Lauschke

**Affiliations:** ^1^ Department of Physiology and Pharmacology and Center for Molecular Medicine Karolinska Institutet and University Hospital Stockholm Sweden; ^2^ Medical Research Center Seoul National University College of Medicine Seoul South Korea; ^3^ Malaria Research and Training Center University of Sciences Techniques, and Technology of Bamako Bamako Mali; ^4^ Dr Margarete Fischer‐Bosch Institute of Clinical Pharmacology Stuttgart Germany; ^5^ University of Tübingen Tübingen Germany; ^6^ Department of Pharmacy, the Second Xiangya Hospital Central South University Changsha China

**Keywords:** Population frequencies, stratified medicine, precision medicine, ethnogeographic variability

## Abstract

Pharmacological responses can vary significantly among patients from different ethnogeographic backgrounds. This variability can, at least in part, be attributed to population‐specific genetic patterns in genes involved in drug absorption, distribution, metabolism, and excretion, as well as in genes associated with drug‐induced toxicity. Identification of such ethnogeographic variability is thus crucial for the optimization of precise population‐specific drug treatments. In this review, we summarize the current knowledge about the clinically actionable pharmacogenetic diversity of genes involved in drug metabolism (*CYP2B6*, *CYP2C8*, *CYP2C9*, *CYP2C19*, *CYP2D6*, *CYP3A5*, *DPYD*, *TPMT*, *NUDT15*, *UGT1A1*, and *NAT2*), drug‐induced hypersensitivity reactions (*HLA‐A* and *HLA‐B*), and drug‐induced acute hemolytic anemia (*G6PD*). We highlight risk populations with distinct allele frequencies and discuss implications for the customization of treatment. Subsequently, we discuss key challenges and opportunities in population pharmacogenomics, including the importance of considering distinct allele frequency patterns in indigenous or founder populations, interpreting pharmacogenomic response in admixed populations, addressing the investigation bias of the pharmacogenomic literature, and difficulties in including rare and population‐specific variants into drug response predictions. The information provided here underscores the critical role of population pharmacogenomics in refining pharmacological treatment strategies and aspires to provide further guidance to maximize the benefits of precision medicine across populations.

## 1 Introduction

Interindividual variability in drug efficacy and safety is commonly observed in pharmacological treatments. It is estimated that only 2%–25% of patients taking the top ten highest‐grossing drugs in the United States in 2015 benefit from treatment (Schork 2015). This variability in efficacy is paralleled by differences in adverse drug reactions (ADRs). ADRs account for an estimated 6%–12% of all hospital admissions (Pirmohamed et al. 2004; Odar‐Cederlöf et al. 2008). Furthermore, ADRs account for 9% of overall healthcare costs (Gyllensten et al. 2014) and postmarket safety events affect approximately one‐third of newly approved therapeutics (Downing et al. 2017).

It is estimated that genetic variations in pharmacogenes are responsible for up to 20%–30% of such variability in drug response (Sim et al. 2013). Due to low selective pressures, many pharmacogenes are highly polymorphic and contain a plethora of single‐nucleotide variants (SNVs), indels and copy‐number variations (CNVs) (Kozyra et al. 2017; Santos et al. 2018). While most functionally characterized variants are missense, that is, result in amino acid exchanges in the polypeptide sequence of the encoded gene product, also an increasing number of stop‐gain, splice, and regulatory variations are identified that cause functional alterations (Tremmel et al. 2023). Importantly, many pharmacogenetic variations exhibit substantial ethnogeographic variability and, consequently, drug‐metabolizing phenotypes can vary significantly between different groups, leading to differences in drug response at the population scale.

In addition to variability in drug metabolizing genes, variants in pharmacodynamic genes as well as in genes encoding the major histocompatibility complex (MHC) are associated with interindividual differences in therapeutic outcomes. For drug targets, this includes rare variants in drug‐binding pockets that can alter binding and the activation of downstream signaling cascades (Hauser et al. 2018; Zhou et al. 2021). For MHC proteins, this includes both rare and common HLA haplotypes, such as HLA‐B*15:02, which is strongly associated with carbamazepine and phenytoin‐induced severe cutaneous adverse reactions, predominantly in Southeast Asian populations where this risk allele can have frequencies over 20%.

In this review, we first provide an updated overview of the ethnogeographic diversity of genetic variations in the most clinically relevant pharmacogenes, including *CYP2B6*, *CYP2C8*, *CYP2C9*, *CYP2C19*, *CYP2D6*, *CYP3A5*, *DPYD*, *TPMT*, *NUDT15*, *UGT1A1*, *NAT2*, *G6PD*, *HLA‐A*, and *HLA‐B*, and discuss their implications for genetically guided drug dosing optimization. Subsequently, we discuss current important opportunities that arise from considering this pharmacogenomic diversity as well as the accompanying technical and methodological challenges. In summary, we conclude that embracing pharmacogenomic diversity is highly important to guide the optimization of population‐specific genotyping strategies and to maximize the benefits of precision public health for diverse patient groups.

## 2 Pharmacogenes

### 2.1 CYP2B6

The *CYP2B6* locus is highly polymorphic with currently 48 defined star alleles (Desta et al. 2021). CYP2B6 is involved in the metabolism of many prescribed drugs including efavirenz, nevirapine, and artemisinin, as well as various environmental pollutants (Hodgson and Rose 2007; Ng et al. 2015). Among all *CYP2B6* alleles that alter gene function, CYP2B6*6 and CYP2B6*9 are overall the most common (Table 1). Both alleles share the splice variant rs3745274 (NM_000767.5:c.516G>T), with *6 additionally containing the missense variant rs2279343 (NM_000767.5:c.785A>G), and both lead to a decrease CYP2B6 activity. CYP2B6*6 is most frequent in Papua New Guinea with minor allele frequencies (MAFs) of 62%, while prevalence in Africa is somewhat lower but, with 21%–47% still considerable (Klein et al. 2005; Li et al. 2012). By contrast, the prevalence of CYP2B6*6 in Europe and Asia is considerably lower with frequencies between 7% in Finland (Li et al. 2012), 16% in Japan (Hiratsuka et al. 2002), 18% in China (Guan et al. 2006), and 23% in Iran (Zakeri et al. 2014). CYP2B6*9 is most common in Asia (4‐10%) except for Japan, where the allele is absent.

**TABLE 1:** Overview of the most important pharmacogenes and their global distribution.

Gene	Most important alleles	Most relevant ancestries	Highest allele frequency and corresponding ancestries	Impacted drugs
*CYP2B6*	CYP2B6*9 CYP2B6*6	Oceania, Sub‐Saharan Africa	14.8%; South America	Efavirenz
		Oceania, Sub‐Saharan Africa	62%; Oceania	
*CYP2C8*	CYP2C8*2 CYP2C8*3	Sub‐Saharan Africa	15.6%; Sub‐Saharan Africa	Aminoquinoleines (amodiaquine, chloroquine, hydroxychloroquine)
		Europe	10.1%; Europe	
*CYP2C9*	CYP2C9*2	Middle East and Europe	13.2%; Europe	Warfarine, NSAID of the oxicam class (e.g., piroxicam, meloxicam, etc), anticonvulsicant (phenytoin)
	CYP2C9*3	Middle East and Europe	11%; Central/South Asia	
*CYP2C19*	CYP2C19*2	Asia and Oceania	40.9%; Oceania	Antiplatelet drugs (clopidogrel), SSRIs (sertraline, fluoxetine)
	CYP2C19*3	Asia and Oceania	8.9%; East Asia	
	CYP2C19*17	Europe	22.1%; Europe	
*CYP2D6*	CYP2D6*4	Europe	16.7%; Europe	Codeine, tamoxifen, risperidone, imipramine and other tricyclic antidepressants
	CYP2D6*5	Sub‐Saharan Africa	6%; Sub‐Saharan Africa	
	CYP2D6*10	East Asia	45.6%; East Asia	
	CYP2D6*17	Sub‐Saharan Africa	18.8%; Sub‐Saharan Africa	
	CYP2D6*29	Sub‐Saharan Africa	10%; Sub‐Saharan Africa	
	CYP2D6*41	North Africa	15.1%; Middle East and North Africa	
*CYP3A5*	CYP3A5*1	Asia, Sub‐Saharan Africa	44.5%; Sub‐Saharan Africa	Tacrolimus
	CYP3A5*3	Europe	93.3%; Europe	
	CYP3A5*6	Europe	17.4%; Sub‐Saharan Africa	
	CYP3A5*7	Europe	9%; Sub‐Saharan Africa	
*DPYD*	c.1905+1G>A (DPYD*2A)	Europe	0.5%; Europe	Fluoropyrimidines (5‐fluorouracil, capecitabine, flucytosine, tegafur)
	*c.2846A>T (D949V)*	Europe	0.6%; Europe	
	c.1679T>G (I560S, DPYD*13)	Europe	0.058%; Europe	
	*c.1129‐5923C>G, c.1236G>A (HapB3)*	Europe	2%; Europe	
*TPMT and NUDT15*	TPMT*3A	Europe and South America	3.8%; South America	Thiopurines (azathioprine, mercaptopurine, thioguanine)
	TPMT*3C	Sub‐Saharan Africa, East, Central and South Asia	5.5%; Sub‐Saharan Africa	
	NUDT15*3	East, Central and South Asia	10.2%; East Asia	
*UGT1A1*	UGT1A1*28	Africa and Central and South Asia	44.8%; Sub‐Saharan Africa	Atazanavir and irinotecan
	UGT1A1*6	East Asia	16.4%; East Asia	
*HLA‐A*	HLA‐A*31:01	Asia and South America	4.3%; East Asia	Carbamazepine
*HLA‐B*	HLA‐B*57:01	Africa	7.9%; Central/South Asia	Abacavir
	HLA‐B*15:02	Asia	2%; Central/South Asia	Carbamazepine, Phenytoin
	HLA‐B*58:01	East and Southeast Asia, Sub‐Saharan Africa	6.2%; East Asia	Allopurinol
*G6PD*	*A‐^202A/376G^*	Central Sub‐Saharan Africa	10‐24%; Sub‐Saharan Africa	Antimalarials (e.g., primaquine and tafenoquine), antibiotics (e.g., dapsone and sulfonamides)
	*A‐^968C/376G^*	West Africa	7‐11%; West Africa	
	Mediterranean, rs5030868	Middle East and Central/South Asia	1‐9%; Middle East and Central/South Asia	

NSAID = non‐steroidal anti‐inflammatory drug; SSRI = selective serotonin reuptake inhibitor.

CYP2B6*6 and CYP2B6*9 have important implications for the clinical therapy with CYP2B6 substrates. Efavirenz is a non‐nucleoside reverse transcriptase inhibitor and a cornerstone of HIV‐1 therapy. The drug exhibits considerable interindividual variability in its pharmacokinetics, which are at least in part due to variations in *CYP2B6*. Specifically, patients homozygous or compound heterozygous for CYP2B6*6 and CYP2B6*9 have significantly reduced efavirenz clearance and are at higher risk of toxicity (Yimer et al. 2012). Consequently, for such poor metabolizers (PMs), lower efavirenz starting doses are recommended (200–400 mg/d instead of 600 mg/d) (Desta et al. 2019). Notably, differences in response between populations remained even after correcting for the CYP2B6*6 genotype, suggesting that further genetic or environmental factors contribute to efavirenz variability (Ngaimisi et al. 2013). When cross‐referencing the facts (i) that 65% of HIV patients reside in Eastern and Southern Africa and (ii) that >25% of individuals in Sub‐Saharan Africa are genetically PMs for CYP2B6, it is easy to appreciate that such genetically guided dosing could have significant clinical impacts in these particularly vulnerable populations.

### 2.2 CYP2C8

CYP2C8 metabolizes more than 100 clinically used drugs, including amodiaquine, cerivastatin, dasabuvir, loperamide, montelukast, paclitaxel, repaglinide, and glitazones, and genetic *CYP2C8* variability can have a considerable impact on enzymatic activity (Backman et al. 2016). Of the more than 700 *CYP2C8* variants identified to date, the most important variant alleles are CYP2C8*2 and CYP2C8*3. In Sub‐Saharan African populations, CYP2C8*2 is the most frequent with MAFs up to 36.9%, whereas CYP2C8*3 is less common (<5%) (Camara et al. 2024). This pattern is inverted in Europe and the Americas where CYP2C8*3 is the dominant variant allele (7%–20%) while CYP2C8*2 is rare (<2%). Similar findings were recently published also for North African populations in Tunisia and Libya (Messaoudi et al. 2024). In East Asia, both CYP2C8*2 and CYP2C8*3 are rare with frequencies <2%. Notably, even between geographically overlapping populations, there can remain large differences. This can be exemplified by the Fulani, Mossi, or Rimaïbe peoples that populate areas across the Sahel from the Atlantic coast to the Horn of Africa. While frequencies of CYP2C8*2 are very high in Mossi or Rimaïbe (23%), they are much lower in the Fulani (9.9%) despite territorial overlap (Camara et al. 2024).

CYP2C8 catalyzes the oxidation of amodiaquine, the main antimalarial used for treatment as well as for seasonal malaria chemoprevention. CYP2C8*2 and, more controversially, CYP2C8*3 have been linked to an increased likelihood of ADRs (Anvikar et al. 2012; Peto et al. 2022). With 249 million cases of malaria and 608,000 deaths (97% of which in Africa), genetically guided dose adjustments of amodiaquine remain of utmost clinical importance. Based on population allele frequencies, the rate of CYP2C8 PMs, as defined here by homozygosity or compound heterozygosity of CYP2C8*2 and CYP2C8*3, is calculated to reach up to 13.6% in African countries, such as the Congo, while the fraction of intermediate metabolizers (IMs) is even higher (up to 46.5%) (Peko et al. 2019). Considering that this region is also an epicenter of malaria, these results suggest that therapeutic drug monitoring during amodiaquine treatment or chemoprevention in genetically at‐risk individuals might be a promising strategy to reduce patient morbidity and increase adherence.

### 2.3 CYP2C9

CYP2C9 is the principal enzyme catalyzing the metabolism of warfarin and various anti‐inflammatory drugs. Globally, CYP2C9*2 and CYP2C9*3 are the most clinically relevant alleles that both lead to decreased CYP2C9 activity (Niinuma et al. 2014). CYP2C9*2 and CYP2C9*3 show similar distribution patterns (Zhou et al. 2023)—both alleles are more prevalent across Europe and the Middle East (8.4%–18.1% for CYP2C9*2 and 4.7%–21.3% for CYP2C9*3) with lower frequencies in Sub‐Saharan Africa (0%–5% for CYP2C9*2 and 0%–3.2% for CYP2C9*3) and East and Southeast Asia (<1% for CYP2C9*2 and 0%–6.6% for CYP2C9*3). An interesting deviation from the convergence of allele frequencies can be found in South Asia, where CYP2C9*2 frequencies are relatively low (1.7%–5%), whereas CYP2C9*3 is highly abundant (9.7%–11.9%). Besides CYP2C9*2 and *3, CYP2C9*5, *6, *8, and *11 are functionally important and can be common, particularly in African populations with frequencies up to 6% (Zhou and Lauschke 2022). At the functional level, these genetic patterns entail that up to 2.3%–11.1% of individuals in Europe and the Middle East are estimated to be CYP2C9 PMs. For such individuals, it is recommended to consider pharmacogenetic algorithms to calculate warfarin dosing (Johnson et al. 2017).

### 2.4 CYP2C19

Like other members of the CYP2C subfamily, CYP2C19 is primarily expressed in the liver but is also found in the duodenum, the small intestine, and, to a lesser extent, in the stomach. In the context of pharmacogenomics, CYP2C19 is of great importance in cardiology and psychiatry. Particularly, its metabolism of antiplatelet drugs, such as clopidogrel, and selective serotonin reuptake inhibitors (SSRIs) such as escitalopram and sertraline is of high clinical relevance. Among the 39 haplotypes described to date, only three are clinically relevant and widely distributed—CYP2C19*2, CYP2C19*3, and CYP2C19*17. CYP2C19*2 and CYP2C19*3 result in a splicing defect and a stop‐gain variation, respectively, rendering the alleles functionally inactive. CYP2C19*2 is common globally with MAFs ranging between 6% in Ghana (Kudzi et al. 2009) and 45% in Papua New Guinea (Masta et al. 2003; Hsu et al. 2008). In contrast to the globally prevalent *2 allele, CYP2C19*3 is restricted to Southeast Asia and the Pacific Islands. However, here frequencies can be very high with MAFs up to 11.5% in Japan (Tsuneoka et al. 1996), 14.4% in Vanuatu (Kaneko et al. 1999), and 19.4% in Papua New Guinea (Ahsen et al. 2010). Consequently, around 15% and 48% of the East Asian population are considered as PMs and IMs for CYP2C19, respectively, entailing an increased risk of clopidogrel treatment failure, manifesting as reduced platelet inhibition and increased risk for major adverse cardiovascular and cerebrovascular events.

In contrast to CYP2C19*2 and *3, CYP2C19*17 constitutes an increased function allele. CYP2C19*17 is highly prevalent in Europe, as well as in Africa and the Americas with frequencies between 10% and 33%. In Asia, CYP2C19*17 is common across South and West Asian populations, such as Iran (21.6%) (Payan et al. 2015)  and India (17%) (Giri et al. 2014). As a result, up to 32% of the population in Europe and Africa is classified as CYP2C19 UMs, whereas this frequency is only 1.8% in East Asia. While UMs do not have altered treatment recommendations for clopidogrel, the available data indicate that UMs have a higher likelihood of not achieving the recommended therapeutic concentrations for escitalopram, resulting in reduced efficacy (Jukić et al. 2018).

### 2.5 CYP2D6

The *CYP2D6* gene is arguably the most polymorphic of all human CYPs with a multitude of common functionally important SNPs, indels, and structural variations described to date. Among the clinically most important haplotypes are CYP2D6*4, *10, *17, *29, and *41. The ethnogeographic variability in this gene has been extensively reviewed, and we refer the reader to these works for details (Gaedigk et al. 2017; Tremmel et al. 2024).

The loss‐of‐function allele CYP2D6*4, characterized by the splice defect variant rs3892097 (NM_000106.6:c.506‐1G>A), has the highest frequencies throughout Europe (MAF 17%–36%) with the exception of Finland, where frequencies are substantially lower (10%). In contrast, in Sub‐Saharan Africa and East Asia, reduced CYP2D6 activity is not primarily attributed to the CYP2D6*4 allele, which only has frequencies of 1%–7% and 0%–3%, respectively. Rather, CYP2D6*10 is the predominant loss‐of‐function allele in Asia with frequencies ranging from 7% in India to 38% in Japan, 47% in China, and 52% in the Philippines. In Sub‐Saharan Africa, CYP2D6*17 (MAF 10%–34%) and CYP2D6*29 (MAF 10%–20%) constitute the most frequent reduced function alleles, which are virtually absent in non‐African populations.

Besides SNV, CNVs are important determinants of CYP2D6 activity. These include *CYP2D6* deletions (CYP2D6*5), as well as functional multiplications of the gene (CYP2D6*1xN and CYP2D6*2xN). For guidelines on how to accurately infer CYP2D6 phenotype from genotype, we refer the interested reader to recent tutorials that discuss how to parse the genetic complexity of this locus (Turner et al. 2023). CYP2D6*5 is most common in African populations with frequencies between 3% in Ethiopia (Aklillu et al. 2002) and 14% in the Xhosa people in South Africa (Wright et al. 2010). Functional gene multiplication is associated with ultrarapid metabolism of CYP2D6 substrates, which is particularly common in Middle Eastern and North African populations with frequencies up to 30% (Aklillu et al. 1996; Alali et al. 2022). Frequencies are lower in Sub‐Saharan African populations (0%–6%) with prevalence widely varying between ethnic groups (Twesigomwe et al. 2023). Frequencies of CYP2D6*1xN and CYP2D6*2xN are graded throughout Europe with the highest prevalence in Southern (3%–6%) Europe, whereas frequencies are lower in Central and Northern Europe (0.3%–4.3%) (Petrović et al. 2020).

CYP2D6 participates in the metabolism of around one‐quarter of all clinically prescribed drugs, including antidepressants, neuroleptics, antiemetics, β‐blockers, anti‐arrhythmics, hormone receptor therapeutics, and antimalarial drugs (Taylor et al. 2020). Genetic variability in CYP2D6 has been robustly associated with differences in the pharmacokinetics, efficacy, or toxicity of a multitude of drugs. Examples include tamoxifen, where *CYP2D6* variants are the only genetic determinant of endoxifen plasma concentrations (Khor et al. 2023), the antipsychotic risperidone, where CYP2D6 genotype significantly correlates with therapeutic failure at standard doses (Jukic et al. 2019), as well as the antiemetic ondansetron, where CYP2D6 UMs are at an increased risk of therapeutic failure due to rapid ondansetron clearance (Kaiser et al. 2002). Thus, *CYP2D6* constitutes one of the currently most actionable loci for pharmacogenetically guided treatment optimization across therapeutic areas.

### 2.6 DPYD

The *DPYD* gene encodes dihydropyrimidine dehydrogenase (DPD), an enzyme responsible for the catabolism of over 80% of fluoropyrimidine drugs, including 5‐fluorouracil (5‐FU) and its prodrugs, capecitabine and tegafur, which are widely used in the treatment of various solid cancers. Genetic variants in *DPYD* can lead to DPD deficiency, impairing drug metabolism and resulting in the accumulation of active drug metabolites. This condition may result in severe toxicities, including myelosuppression, mucositis, and hand–foot syndrome that can even be fatal (Caudle et al. 2013). Partial DPD deficiency is estimated to occur in 3%–5% of the Caucasian population, while complete deficiency has an estimated prevalence of 0.1%–0.3% (Etienne‐Grimaldi et al. 2023).

*DPYD* is a highly polymorphic gene, with over 20 loss‐of‐function variants identified that contribute to variability in enzyme activity and drug metabolism (Larrue et al. 2024). Among these, the most clinically significant variants are c.1905+1G>A (DPYD*2A), c.2846A>T (p.D949V), c.1236G>A (HapB3), c.1679T>G (p.I560S, DPYD*13), and c.1129‐5923C>G (Meulendijks et al. 2015). DPYD*2A results in complete loss‐of‐function due to exon skipping, while c.1679T>G and c.2846A>T lead to reduced enzyme activity via deleterious amino acid substitutions and an intronic variant in HapB3 reduces expression via introduction of a cryptic splice site.

The allele frequencies of these variants exhibit marked variability across ethnic groups. DPYD*2A is most common in European populations with frequencies up to 2.4% in Finland, but it is exceedingly rare in Asian and African populations (Zhou et al. 2020). Similarly, c.2846A>T is observed in 1.1% of Caucasians but occurs at lower frequencies in African and Asian populations (0.1%) (Offer et al. 2014). These population‐specific differences highlight the need for tailored population‐agnostic strategies to ensure the safe and effective use of fluoropyrimidines.

To mitigate the risk of severe toxicity, preemptive *DPYD* genotyping is now mandated in the United Kingdom and Europe (Chan et al. 2024). Mandatory screening focuses on four key variants (DPYD*2A, c.2846A>T, DPYD*13, and c.1236G>A/HapB3), which are estimated to predict 20%–30% of early‐onset life‐threatening 5‐FU toxicities (NHS, UK 2020). This approach is supported by evidence that dose reductions of up to 50% for individuals with heterozygous genotypes can maintain therapeutic efficacy while mitigating the risk of severe toxicity (Henricks et al. 2018). Although the implementation of *DPYD* genotyping is less common in populations with lower variant frequencies, such as East Asians, alternative strategies, including therapeutic drug monitoring or dose adjustments based on observed toxicities, may provide practical benefits in these settings (Kanai et al. 2023). The incorporation of *DPYD* genotyping into routine oncological practice underscores the critical role of pharmacogenomics in optimizing cancer care. By identifying patients at risk for severe fluoropyrimidine toxicity, healthcare providers can make more informed dosing decisions, improving both the safety and efficacy of these life‐saving therapies.

### 2.7 TPMT and NUDT15

Thiopurine drugs are cornerstone therapies for a variety of conditions, including acute lymphoblastic leukemia, inflammatory bowel disease, and autoimmune disorders. The metabolism and detoxification of these drugs depend on thiopurine S‐methyltransferase (TPMT) and nudix hydrolase 15 (NUDT15). These enzymes regulate the production and detoxification of cytotoxic thioguanine nucleotides (TGNs), which are responsible for both the therapeutic and adverse effects of thiopurines. Deficiencies in either enzyme can lead to an accumulation of TGNs, significantly increasing the risk of severe toxicities, particularly myelosuppression.

Genetic variations in *TPMT* and *NUDT15* are well‐documented determinants of thiopurine metabolism. The most clinically significant variants in TPMT include TPMT*2, TPMT*3A, and TPMT*3C. Among these, TPMT*3A is the most prevalent in individuals of European descent (3.8%) and Latinos (4.3%), while TPMT*3C is more common in Asians (1.1%–1.4%) and Africans (4.8%) (Zhou et al. 2020). For *NUDT15*, c.415C>T (p.R139C, rs116855232) has emerged as a major pharmacogenomic marker, accounting for 22% of the variability in thiopurine tolerance (Moriyama et al. 2016). This variant is most prevalent in East Asians, with allele frequencies exceeding 10%, but is rare in Latino and European populations, where it occurs at less than 1% (Singh et al. 2017). Beyond these common variants, additional rare *NUDT15* variants, such as p.V18I and p.G17_V18del, have been identified, though their impact is less well characterized.

Pharmacogenomic guidelines provide strong recommendations for dose adjustments based on *TPMT* and *NUDT15* genotypes (Relling et al. 2019). TPMT*2, TPMT*3A, TPMT*3C, and *NUDT15* c.415C>T are moreover recommended by the Association for Molecular Pathology for preemptive genotyping to guide dosing in higher‐risk populations (Pratt et al. 2022). These TPMT variants account for 90% of reduced enzyme activity phenotypes in Europeans, while NUDT15 c.415C>T represents over 50% of the cataloged alleles in the general population. CPIC guidelines recommend thiopurine dose reductions of 85%–90% for individuals with homozygous deficiencies of TPMT or NUDT15, and of 30%–70% for IMs (Relling et al. 2019). These evolving guidelines continue to prioritize patient safety and therapeutic efficacy across diverse populations.

### 2.8 UGT1A1

The *UGT1A1* gene encodes the key enzyme responsible for the glucuronidation and elimination of bilirubin and various drugs, including the chemotherapeutic agent irinotecan. The most clinically relevant variants are related to an indel variation in the *UGT1A1* promoter region that impacts gene expression (rs34983651). While the reference allele contains six TA repeats, UGT1A1*28 and UGT1A1*37 contain seven and eight repeats, respectively, which reduces transcriptional efficiency (Beutler et al. 1998). UGT1A1*80, a synonymous variant often co‐inherited with UGT1A1*28 due to linkage disequilibrium, may also indirectly influence enzyme function. These variants are associated with mild hyperbilirubinemia and impaired irinotecan clearance. Of additional relevant is the missense variant p.G71R (UGT1A1*6), which is associated with decreased enzyme function.

UGT1A1*28 is most prevalent in African populations (43%), followed by Europeans (39%) and Asians (16%), whereas UGT1A1*6 is almost exclusively found in East Asian populations, occurring at frequencies of 13% in Japanese, 23% in Korean, and 23% in Chinese, while being rare in Caucasians (1%) and Africans (0.1%) (Akaba et al. 1998; González‐Padilla et al. 2024). Similarly, UGT1A1*37 is most frequent in Europeans (1%–5%) and Africans (2%–7%), while being virtually absent in Asians.

Individuals homozygous or compound heterozygous for reduced function *UGT1A1* alleles are at increased risk of severe irinotecan‐induced toxicities at standard doses. Pharmacogenomic guidelines therefore recommend dose adjustments based on *UGT1A1* genotypes to mitigate these adverse effects (Hulshof et al. 2023). Similar recommendations are in place for atazanavir (Gammal et al. 2016). Implementation of *UGT1A1* genotyping into clinical practice thus constitutes a potential strategy to enhance the safety and efficacy of irinotecan and atazanavir therapy.

### 2.9 NAT2

The *NAT1* and *NAT2* genes encode enzymes responsible for the acetylation of various nitrogenous compounds, including the antimycobacterial drug isoniazid, which is the primary treatment for tuberculosis. Compared with *NAT1, NAT2* is more functionally polymorphic and exhibits significant genetic variability across different ethnogeographic groups. NAT2*4 is the principal haplotype resulting in fast metabolism, while NAT2*5, *6, and *7 result in slow metabolism. Among these, NAT2*5 is most common with frequencies up to 58% in Caucasians, whereas its prevalence in Asian populations is considerably lower, pivoting around 5% (Sabbagh et al. 2011). In contrast, NAT2*7 is relatively rare among Caucasians (<5%) but is more common in Asians (10%–20%). Such a functional diversity has also been observed in populations within admixed groups, leading to heterogenous acetylation profiles (Lopes et al. 2023).

Slow acetylator phenotypes have been consistently associated with an increased risk of liver injury due to antituberculosis therapy. Notably, odds ratios differed between ethnic groups with the highest risk being identified for West Asian populations with an odds ratio (OR) of 6.4, whereas risk in South and East Asia was lower with ORs between 2.3 and 2.4 (Zhang et al. 2018; Mahajan and Tyagi 2024). Despite this evidence, it currently remains unclear whether NAT2 genotyping should be recommended prior to anti‐tuberculosis treatment.

### 2.10 HLA genes

Unlike pharmacogenes that are involved in either pharmacokinetic or pharmacodynamic processes, the human leukocyte antigens (HLAs) genes can interact with drugs in an off‐target and mostly dose‐independent manner, which, as a result, can trigger immunologically mediated idiosyncratic drug reactions (IDRs). These IDRs include severe adverse reactions, such as Stevens‐Johnson syndrome (SJS) and toxic epidermal necrolysis (TEN) with mortality rates up to 60% (Schulz et al. 2000; Watanabe et al. 2021). Different mechanisms have been suggested for how genetic variations in HLA molecules can trigger immune activation (Deshpande et al. 2021). Drug molecules can change the conformation of peptide‐binding sites in the major histocompatibility complex by directly binding in a covalent manner to presented peptides (the hapten model). Alternatively, they can non‐covalently bind to the HLA molecule or T‐cell receptor (the pharmacological interaction model). Lastly, they can covalently bind to HLA molecules and alter the presented peptide repertoire (the altered peptide model).

*HLA* genes are extremely polymorphic, with more than 35,000 alleles being described to date (Barker et al. 2022). However, only around a hundred of those have been associated with IDRs. The most well‐established risk alleles are HLA‐B*57:01 for abacavir hypersensitivity syndrome (ABC‐HSS), HLA‐B*15:02 and HLA‐A*31:01 for carbamazepine‐induced SJS/TEN, and HLA‐B*58:01 for allopurinol‐induced SJS/TEN or drug rash with eosinophilia and systemic symptoms (Pavlos et al. 2015). Genotyping for these HLA alleles can reliably identify patients at risk with a high negative predictive value of 99.9%–100%. In contrast, the positive predictive value (PPV) is typically considerably lower (<50% but can be as low as 1%). This means that only carriers of the respective alleles are at risk of experiencing the IDRs while non‐carriers are fully protected. However, among the risk allele carriers, only a small fraction of exposed patients will experience clinically manifest adverse events.

**FIGURE 1:**
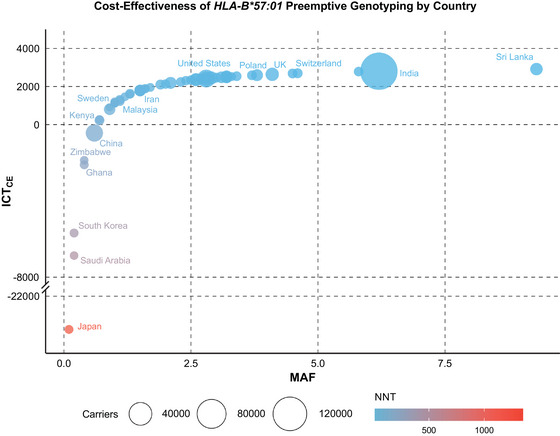
Cost‐effectiveness of preemptive HLA‐B*57:01 genotyping across countries. The size of the dots represents the number of allele carriers (in thousands), while the color of each dot corresponds to the number of patients needed to test to prevent one case of adverse event (NNT) for each country. The incremental cost threshold for cost‐effectiveness (ICT_CE_) indicates the threshold value for a “willingness to pay” threshold (ICER_T_) of USD 40,000. The plot has been modified based on data from Zhou et al. (2021). MAF = minor allele frequency.

Among the well‐established clinically actionable HLA associations, the HLA‐B*57:01‐abacavir gene–drug pair has the highest PPV of 47.9% (Mallal et al. 2008). Combined, with the relatively high allele frequencies of 3%–5% in Europe and up to 8%–10% in India and Sri Lanka, preemptive typing of HLA‐B*57:01 to avoid ABC‐HSS is mostly cost‐effective (Zhou et al. 2021). Consequently, both the U.S. Food and Drug Administration (FDA) and European Medicines Agency have mandated the testing of HLA‐B*57:01 prior to initiating or reinitiating abacavir treatment. However, in countries like South Korea, Saudi Arabia, and Japan where HLA‐B*57:01 allele frequencies are very low (<1%), the incremental cost‐effectiveness threshold (ICT_CE_) is negative (Figure 1), indicating that the cost of preemptive genotyping is not a cost‐effective allocation of healthcare resources.

In contrast to HLA‐B*57:01, HLA‐B*15:02 is almost exclusively found in Southeast Asia with MAFs up to 22% in the Philippines (Tremmel et al. 2024), whereas frequencies are considerably lower in East and South Asian countries, such as China (3.8%), India (1.9%), and Japan (0.1%). In populations of European, African, or American ancestry, HLA‐B*15:02 is absent. HLA‐B*58:01 is similarly population‐specific. It is common across Asia and Africa (1.7%–8.8%) but rarely found in European and North American populations (<1%). Notably, both alleles are highly specific risk alleles but have low PPVs (5.6% for HLA‐B*15:02 for carbamazepine and 1.3% for HLA‐B*58:01 for allopurinol). Consequently, population differences in frequency directly impact the economic viability of testing. For instance, less than 85 patients initiating carbamazepine therapy would need to be tested for HLA‐B*15:02 to avoid one case of SJS/TEN in the Philippines, Malaysia, Indonesia, Singapore, and Vietnam, whereas > 9,000 individuals need to be tested to prevent one case in Europe (Zhou et al. 2021). Combined, these significant ethnogeographic differences in *HLA* alleles frequencies thus showcase the important implications for informing IDR risks across populations.

### 2.11 G6PD

*G6PD* encodes glucose‐6‐phosphate dehydrogenase, an enzyme that catalyzes the oxidation of glucose‐6‐phosphate coupled to the reduction of NADP to NADPH, which is an important electron donor that can protect cells from oxidative stress (Luzzatto et al. 2020). G6PD deficiency is the most common hereditary monogenic defect affecting an estimated 500 million people worldwide. While mostly asymptomatic, exposure to different dietary components like fava beans or medications such as antimalarials (e.g., primaquine and tafenoquine) or antibiotics (e.g., dapsone and sulfonamides) can trigger life‐threatening acute hemolytic anemia (Youngster et al. 2010). *G6PD* is highly polymorphic, with more than 1,300 variants identified to date, of which more than 250 are classified as causing reduced G6PD activity (Geck et al. 2023). Notably, *G6PD* is located on the X‐chromosome; therefore, G6PD deficiency is predominantly found in hemizygous males (Domingo et al. 2019).

G6PD deficiency is most common in Sub‐Saharan Africa, followed by the Middle East and South Asia (Nkhoma et al. 2009). However, there can be striking differences at the regional level, as exemplified by frequencies of up to 29% in Gabon, whereas G6PD deficiency is very rare in the South of the continent and the Horn of Africa (Howes et al. 2013). The frequencies of the underlying molecular basis also exhibit distinct ethnogeographic patterns. The most frequent G6PD‐deficient variants A‐^202A/376G^ and A‐^968C/376G^ have average frequencies of 10%–24% and 1% in Sub‐Saharan Africa, respectively. However, frequency profiles are inverted among West African populations with the allele A‐^968C/376G^ allele being more common (7%–11%) than A‐^202A/376G^ (1%–3%) (Koromina et al. 2021). In non‐African, the two A‐ alleles are rare, and G6PD deficiency is largely determined by other alleles: in the Middle East, the Mediterranean (rs5030868; p.S188F), Cairo (rs782322505; p.N135T), and Chatham (rs5030869; p.A335T) variants are most impactful (Alfadhli et al. 2005; Al‐Allawi et al. 2010), while the Mediterranean and Orrisa variants account for 24% and 57% of G6PD deficiency in India, respectively (Devendra et al. 2020). In Southeast Asian populations, the dominant variants are Viangchan (rs137852327; p.V291M) and Mahidol (rs137852314; p.G163S) (Tantular and Kawamoto 2021), whereas Kaiping (rs72554664; p.R463H) and Canton (rs72554665; p.R459L) are most frequent in Han Chinese (He et al. 2020). Overall, these data indicate that public health strategies using antimalarials associated with a risk of hemolytic anemias need to consider population‐specific patterns of pharmacogenetic variability to tailor genotyping and treatment strategies and minimize patient morbidity.

## 3 Challenges and Opportunities

Pharmacogenetic diversity has a substantial impact on drug response phenotypes. A multitude of studies of the last decades revealed that the underlying molecular basis can differ drastically between ethnogeographic groups. For instance, the CYP2C19*3 allele, the presence of which is a counterindication for the use of clopidogrel, shows significant variability across geographically adjacent populations. While this allele is relatively prevalent in Japanese (9%) and Koreans (10.1%), it is nearly absent in South Asians (<1%), suggesting that testing in the context of clopidogrel prescribing is only meaningful in populations in which the allele is prevalent (Fricke‐Galindo et al. 2016). Furthermore, a recent study suggested based on analyses of whole‐genome sequencing data sets that genetic ancestry is correlated with the overall risk of drug toxicity, suggesting further opportunities to optimize precision pharmacotherapy (Karamperis et al. 2024). In recognition of these differences in functional allele frequencies, ancestry information is recommended to be used by various guidelines to stratify if patients should be tested and, if so, which variants should be probed. One example of such a guideline is the FDA‐approved label for carbamazepine, which states that HLA‐B*15:02 should be tested for prior to initiation of carbamazepine therapy specifically in Asian patients. Similarly, the American College of Rheumatology recently recommended that testing for the HLA‐B*58:01 allele prior to starting allopurinol is conditionally recommended for African American patients and patients of South and East Asian descent, whereas testing is recommended against in all other ethnic groups (Fitzgerald et al. 2020).

While patient stratification based on ethnogeographic information constitutes a step in the right direction to offer the best evidence‐based treatment, such recommendations still suffer from multiple shortcomings. Firstly, aggregation into macropopulations often does not have the required resolution to provide the best decision support as summarized by a recently published pharmacogenetic allele frequency database (PharmFreq) that aggregated data from >1,200 studies (Tremmel et al. 2024). For instance, HLA‐B*15:02 frequencies showed drastic differences across Asia with high frequencies in Burma, Indonesia, Vietnam, and the Philippines (>10%), whereas prevalence was substantially lower in China and India (3.8% and 1.9%, respectively), and this allele was almost absent in South Korea and Japan (<0.5%). Furthermore, frequencies can have marked geographical differences even in relatively homogeneous cohorts, as exemplified by the case of Switzerland where HLA‐B*58:01 frequencies in the city of Basel were reported to be over four‐times higher than in Bern, only 100 km away (Goodman and Brett 2021). Regional initiatives illustrate how localized efforts can drive the practical implementation of pharmacogenomics while addressing disparities in access and adoption. In Spain, different regions like Madrid, Castilla y León, Extremadura, and Galicia have made significant progress in integrating pharmacogenomics into clinical practice. By addressing regional healthcare priorities, these initiatives ensure that pharmacogenetic assays remain clinically relevant and in line with recommendations from national and international pharmacogenetic consortia and databases. Furthermore, alignment of regional efforts under a unified national framework fosters a collaborative approach to precision medicine that ensures that advancements in pharmacogenomics benefit all regions across Spain.

Importantly, however, while approaches with higher geographical resolution can improve local decision‐making, the translation of such information into clinical practice remains challenging. Do pharmacogenomic initiatives need to issue guidelines at the level of region or at the level of ancestry or both? Should frequency thresholds be defined for regional populations above which genetic testing is recommended? If the latter is advocated for, current guidelines that recommend, e.g., “testing for HLA‐B*15:02 […] in patients with ancestry in populations in which HLA‐B*15:02 may be present” is not sufficiently concrete since it cannot be left to the treating physician to determine the ancestry of a given patient with sufficient accuracy to make informed decisions about ethnogeographically stratified pharmacogenetic genotyping strategies.

This problem is further amplified in highly heterogeneous populations, such as in Brazil, with pronounced admixture between Amerindians, Europeans, and Sub‐Saharan Africans. While ancestry is typically self‐reported based on the stratification into “White”, “Brown,” and “Black” in the Brazilian census, the genetic landscape can vary drastically within each of these groups. Particularly, individuals identifying as brown or black can range from 100% African ancestry to over 90% European ancestry (Suarez‐Kurtz 2010). Consequently, frequencies of clinically important pharmacogenetic variants cannot be sufficiently captured, rendering the current categorization system clinically not actionable.

**FIGURE 2:**
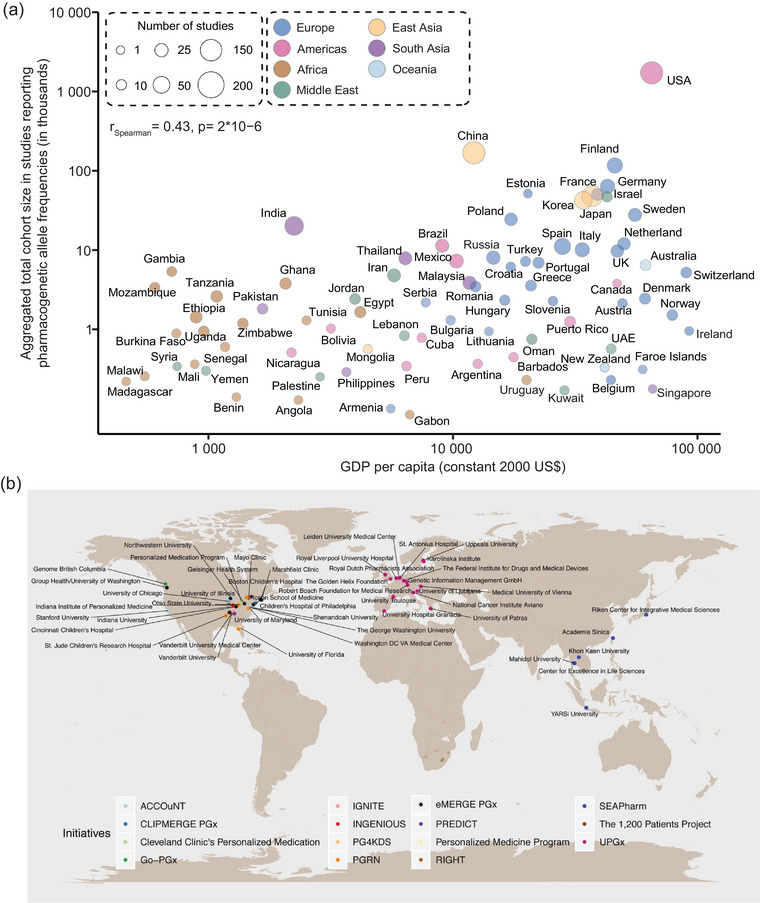
**Global overview of pharmacogenomic studies and implementation initiatives. (A)** Number of pharmacogenomic studies, aggregated cohort sizes, and gross domestic product (GDP) for countries with available pharmacogenomic information. The plot has been modified based on data from Tremmel et al. (2024). **(B)** Names and locations of major pharmacogenetic studies and implementation initiatives (up to 2019). The map was obtained with permission from Krebs and Milani (2019).

While the importance of pharmacogenomics has been recognized globally, the investigated cohorts are unequally distributed across populations. Most studies have focused on European and increasingly Asian populations, primarily due to their higher research budget and more advanced infrastructure (Figure 2A). Furthermore, biobanks that allow for the coupling of large‐scale genomic data with electronic medical records, such as the All of Us Research Program (The All of Us Research Program Investigators 2019), the UK Biobank (Bycroft et al. 2018), and the Estonian Biobank (Reisberg et al. 2019), predominantly contain information from Caucasian participants. Furthermore, most ongoing pharmacogenomic implementation initiatives are found in the United States and Europe with limited representation of South America or Africa (Figure 2B). To bridge this gap, the Human Heredity and Health in Africa (H3Africa) has established large‐scale genomic data sets across diverse African populations to inform decision‐making in African healthcare contexts (H3Africa Consortium 2014). Similarly, the Qatar Genome Programme integrates genomic and clinical data from Middle Eastern populations and has already identified population‐specific variants in *CYP2C19* and *SLCO1B1* that influence responses to antiplatelet drugs and statins with implications for addressing the high burden of cardiovascular diseases in the region (Abdel‐Latif et al. 2024; Elfatih et al. 2024).

Besides the global differences in population coverage, there remains a systematic paucity of studies covering indigenous populations. These groups often possess unique genetic structures primarily due to geographical or cultural isolation that can result in atypical pharmacogenetic profiles. For example, the Jahai people in Malaysia and Thailand, as well as the Tiwi people in the Northern Territory of Australia feature CYP2C9*3 allele frequencies of up to 36%, which is more than ten‐fold higher than in geographically adjacent populations (Rosdi et al. 2016, Shankar et al. 2022). These results suggest that members of these populations are at considerably higher risk of adverse events when treated with warfarin or phenytoin. We thus advocate for an increased inclusion of indigenous populations in pharmacogenomic investigations and initiatives.

Finally, it is important to be aware that the vast majority of pharmacogenetic variants are rare (MAF < 1%) and population‐specific (Ingelman‐Sundberg et al. 2018; Schärfe et al. 2017; Wright et al. 2018). Integration of these variants into pharmacogenetic variability profiles by introducing different sequencing strategies, such as target sequencing, whole‐exome sequencing, or even whole‐genome sequencing, is important to fully translate genetic data into drug response phenotypes. However, the clinical application of sequencing‐based pharmacogenomics still faces multiple challenges, including the cost‐effectiveness and the functional interpretation of novel and rare genetic variants. While sequencing may be cost‐effective in certain therapeutical areas, such as in the diagnostics of neurological disorders (Schwarze et al. 2018) and for informing cancer therapy based on somatic variations (Krebs et al. 2024), the cost‐effectiveness of most germline pharmacogenomic sequencing has not been demonstrated. For interpreting sequencing‐derived rare variants, their low frequencies render epidemiological attempts at functional characterization virtually impossible. Similarly, experimental in vitro strategies are typically impractical for the large numbers of tens of thousands of variations. To overcome these problems, considerable advances in computational variant effect prediction methods have been made, and we refer interested readers to our recently published reviews on this topic (Tremmel et al. 2023; Zhou and Lauschke 2024). With reducing sequencing costs and further advancements in AI, it will be interesting to see how computational tools might further improve personalized drug response predictions based on comprehensive pharmacogenomic data in the near future.

## 4 Conclusion

Ethnogeographic differences in the frequency and distribution of pharmacogenetic variants have been increasingly explored over the past decades. Populations with high frequencies of certain pharmacoalleles may be more susceptible to a lack of efficacy or ADRs when treated with drugs associated with the respective genes. While many advancements have been made in inferring pharmacological phenotypes from pharmacogenetic diversity, their translation into actionable clinical recommendations remains challenging. Pharmacogenetic risk alleles can vary drastically in prevalence not only between geographical areas but also between adjacent, sometimes overlapping populations. Furthermore, inaccuracies connected to the self‐reporting of ancestry and admixture can complicate the consideration of information about population‐based differences. Therefore, how and to what extent patients can be stratified based on pharmacogenetic precision public health strategies, remains a pressing question. In addition, the majority of genomic and pharmacogenomic projects currently focus on European and Asian populations, potentially biasing data interpretation and decision‐making. In particular, indigenous populations who often have unique genetic compositions are commonly underrepresented in pharmacogenomic research. Thus, while the increasing appreciation of pharmacogenomic population variability is commendable, there remains much work to be done to fully leverage the potential of population pharmacogenomics to guide precision medicine and precision public health.

## Author Contributions

All authors contributed to the writing and review of the manuscript. All authors agree to the publication of the accepted version.

## Conflicts of Interest

VML is CEO and shareholder of HepaPredict AB, as well as co‐founder and shareholder of Shanghai Hepo Biotechnology Ltd.

## Data Availability

All reanalyzed data is available in the respective references.
